# Zero Liquid Discharge of High-Salinity Produced Water via Integrated Membrane Distillation and Crystallization: Experimental Study and Techno-Economic Analysis

**DOI:** 10.3390/membranes15090281

**Published:** 2025-09-19

**Authors:** Gabriela Torres Fernandez, Zongjie He, Jeremiah Kessie, Jianjia Yu

**Affiliations:** 1 Department of Civil and Environmental Engineering, New Mexico Institute of Mining and Technology, Socorro, NM 87801, USA; 2 Department of Materials Engineering, New Mexico Institute of Mining and Technology, Socorro, NM 87801, USA; 3 Department of Chemical Engineering, New Mexico Institute of Mining and Technology, Socorro, NM 87801, USA; 4 Petroleum Recovery Research Center, New Mexico Institute of Mining and Technology, Socorro, NM 87801, USA

**Keywords:** membrane distillation crystallization, produced water, salt recovery, zero liquid discharge, economic analysis

## Abstract

Direct Contact Membrane Distillation–Crystallization (DCMD-Cr) is a synergistic technology for zero liquid discharge (ZLD) and resource recovery from high-salinity brines. In this study, DCMD-Cr was integrated to desalinate real oilfield-produced water (PW) with an initial salinity of 156,700 mg/L. The PW was concentrated to its saturation point of 28 wt.% via DCMD, and the integrated crystallization increased the overall water recovery from 42.0% to 98.9%, with a decline in water flux and salt rejection, mainly due to vapor pressure lowering and scaling. The precipitated salts in the crystallization unit were recovered and identified using different techniques. The results indicated that 91% of the crystals are sodium chloride, and less than 5% are calcium sulfate. A techno-economic analysis (TEA) was performed to evaluate the economic feasibility of the integrated DCMD-Cr process with a 500,000 gallons per day (GDP) capacity. The results showed that the crystallization operating cost was dominant at USD 0.50 per barrel, while the capital cost was only USD 0.04 per barrel. The economic viability can be enhanced by recovering value-added byproducts and using renewable or waste heat, which can reduce the total cost to USD 0.50 per barrel.

## 1. Introduction

The production of oil and shale gas is associated with large volumes of produced water (PW) with high salinities, with byproducts rich in hydrocarbons, heavy metals, and organic matters. Around 250 million barrels of PW are produced each day around the world, while the corresponding oil production is 80 million barrels [1]. The disposal of PW raises significant environmental concerns and challenges for the oil and gas industry [2,3]. For instance, PW contains high concentrations of total dissolved solids (TDS), which can reach up to 300,000 mg/L in conventional wells [4]. These high TDS levels pose significant challenges for conventional water treatment methods, as they impair efficiency, cause corrosion and scaling of the equipment, and result in high operational costs. Excessive salinity can also inhibit the growth and metabolic activity of the microorganisms essential for biological treatments. The traditional physical and chemical treatment methods face difficulties related to the cost and management of chemical consumption, byproducts, waste salt discharge/purification after PW treatment, scaling, and low energy efficiency [5]. To address these challenges, zero liquid discharge (ZLD) shows promising potential in the treatment of highly saline PW. ZLD also offers opportunities to simultaneously recover valuable minerals from the concentrated stream, which significantly compensates for the cost for PW treatment [6,7].

Membrane-based desalination processes offer numerous benefits such as high scalability, small carbon footprint, and lower pumping and capital requirements [8]. Specifically, direct contact membrane distillation (DCMD) is an effective process for high-salinity PW desalination, based on a temperature gradient between the hot feed and cold permeate sides. It can theoretically achieve a near-100% salt rejection by only allowing vapor to pass through a hydrophobic microporous membrane [2]. DCMD has received increasing attention due to its lower sensitivity to the salinity of the feed solution, a near-100% salt rejection efficiency, and ease of integration with renewable or low-grade energy.

One of the major challenges associated with DCMD is the accelerated membrane scaling that occurs with increased water recovery as the feed solution approaches its saturation point, resulting in significant declines in water flux and salt rejection. Additionally, the discharge of large amounts of the concentrate stream causes not only the waste of minerals but also significant environmental and health risks [9,10]. To address these challenges, the integration of crystallization into DCMD prevents the feed solution from reaching its saturation point by the continuous removal of concentrated salts from the feed solution [11]. During a membrane distillation and crystallization (MD-Cr) process, high-salinity brine is concentrated to a near-saturation point via MD, and the concentrated salts are recovered in a crystallizer. Standalone brine crystallizers such as the Mixed-Suspension, Mixed-Product-Removal (MSMPR) with evaporative forced-circulation or Draft Tube Baffle (DTB) designs can recover salts without using membranes. These units handle very high TDS but require sizable thermal duty and often suffer from wall scaling and broad crystal-size distributions. Crystallization is governed by primary nucleation (formation of the first nuclei in the absence of crystals, homogeneous/heterogeneous on surfaces at high supersaturation) and secondary nucleation (generation of new nuclei by contact/attrition/seed in a slurry). Intensified concepts such as solid hollow fiber cooling crystallization (SHFCC) increase primary nucleation via distributed cold surfaces, yielding finer halite and mitigating caking [12,13]. Compared to the standalone brine crystallization, the membrane acts as an accelerator and promoter for the crystallization process through secondary nucleation, which significantly reduces the energy demand by simultaneously dewatering the feed solution via membrane distillation [10,14,15].

In recent years, MD-Cr has been reported for both desalination and minerals recovery from various water sources, such as seawater [16,17,18,19,20], RO/NF brines [20,21,22], mining and radioactive wastewater [23,24,25,26,27], and high salinity synthetic brines (e.g., NaCl) [16,28,29,30,31,32,33,34]. RO/NF was typically used to desalinate wastewater with a low-to-moderate salinity (<70,000 mg/L), but was impractical when used to treat wastewater with a high TDS due to elevated osmotic pressure and its associated energy consumption. MD tolerates high salinity but still suffers flux loss when reaching the saturation point. By coupling MD with crystallization, the dissolved salts are continuously removed via crystallization as they became enriched during MD, enabling a stable desalination performance. It was found that the integrated MD-Cr process shows an up to 80–95% water recovery and near-100% salt recovery, which are significantly higher than that of the standalone membrane distillation and crystallization processes [35,36]. However, as summarized in Table 1, there are very few studies on real-produced water desalination using MD-Cr. In 2015, Ali et al. demonstrated a MD-Cr process and obtained high-quality NaCl crystals from oilfield PW with a 37% overall water recovery [37]. Later, another study reported both experimental data and thermodynamic models to demonstrate the capability of recovering barium from oilfield PW though a cooling-based MD-Cr process [11]. According to Kim et al. [38], Salmon et al. [31], and Lu et al. [39], the hybrid MD-Cr process demonstrates high NaCl rejection, effective extraction of various organic salts, and improved water recovery rates due to reduced ion strength in the feed solution. These results were obtained by simulating ternary oil extraction wastewater, synthetic flue gas wastewater, and synthetic-produced water (PW), respectively. However, the economic potential for both water and mineral-valued products and recovery rates was not specified in these previous studies [40,41,42].

In this study, an integrated DCMD and Crystallization (DCMD-Cr) process is developed to desalinate actual oilfield-produced water. The produced water was concentrated to the point close to saturation through DCMD; the salts in the concentrated produced water were recovered in a crystallizer, and the remaining produced water was sent back to the DCMD unit for a near-100% water recovery. Long-term DCMD-Cr experiments were conducted to assess the membrane’s long-term stability until reaching zero liquid discharge (ZLD). A techno-economic analysis (TEA) was performed to evaluate the economic feasibility of the DCMD-Cr process for the simultaneous recovery of water and salts from the produced water.

## 2. Methods

### 2.1. Materials and Characterization

#### 2.1.1. Materials

Produced water was sampled from Southeast New Mexico in the Permian Basin. The initial PW TDS was 157,000 ppm, with the major cations and anions listed in Table 2. The original PW was pre-treated with a 50 µm cartridge filter without the use of any chemicals.

The TDS were measured with a portable multi-parameter meter (HQ40d, Hach, Loveland, CO, USA); cations and anions were measured via ion chromatography (Dionex ICS-1100, Thermo Scientific, Sunnyvale, CA, USA). The morphology of membranes was examined using a field emission scanning electron microscope (JEOL JSM-IT700HR, Peabody, MA, USA). The elemental composition and distributions were obtained using SEM-mounted Energy Dispersive Spectroscopy (EDS) through mapping and point analysis.

#### 2.1.2. Membrane Modules Preparation

A home-made PVDF hollow fiber membrane was used to assemble the membrane modules. Each membrane module contains six hollow fibers with a length and surface area of 22 cm and 45.10 cm^2^ (based on outer diameter), respectively. The main properties of the membrane are summarized in Table 3 according to a previous article [44].

### 2.2. Direct Contact Membrane Distillation and Crystallization (DCMD-Cr)

**Figure 1:**
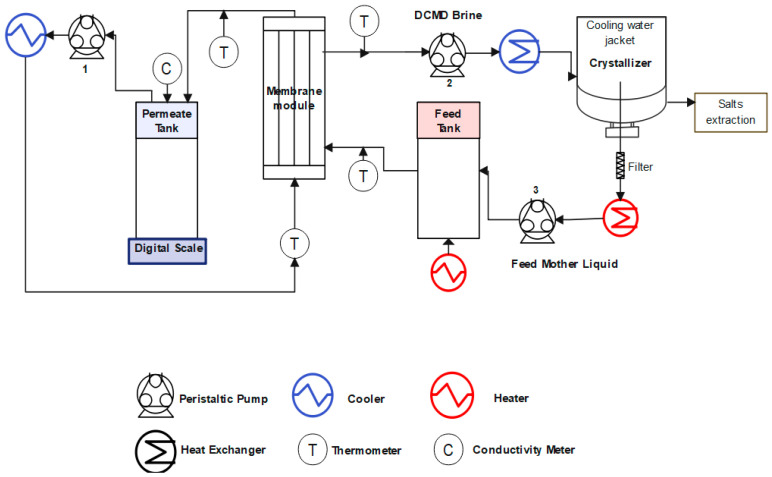
Schematic of the integrated DCMD-Cr process for ZLD of PW.

The apparatus for the membrane distillation and crystallization experiment is demonstrated in Figure 1. The specific membrane surface area was 45.10 cm^2^. For all the experiments, PW was pre-filtered with a 50 µm filter. During membrane distillation, the pre-filtrated produced water (4.0 L) was heated to 60 °C and flowed through the shell side of the PVDF hollow fiber membranes; the flow rate was 0.7 m/s. Deionized water (20 °C) was circulated on the permeate side, and the flow rate was 0.3 m/s. The temperature at both inlets and outlets of the membrane module were monitored by four online temperature transducers. The weight of the permeate water was monitored by a digital balance equipped with a data acquisition system. The concentrated produced water was sent to a crystallizer (5 °C) and returned back to the feed tank for future water recovery. During the DCMD-Cr experiments, the permeate water flux and electrical conductivity were continuously monitored every 5 min to estimate water recovery and salt rejection. The permeate water flux is calculated using Equation (1), while the salt rejection can be obtained using Equation (2).

(1) $$J=\frac{M}{A\times t}$$

(2) $$R=\left(1-\frac{C_{permeate}}{C_{feed}}\right)\times 100\%$$

where *M* is the permeate water mass in kg, *A* is the total effective surface area of the hollow fiber membranes in m^2^, and *t* is the operating time in hours; $C_{permeate}$ and $C_{feed}$ are the salt concentrations in the permeate and feed solution, respectively.

The precipitated salts in the crystallizer were collected and dried at 85 °C in a vacuum oven. The dry crystals were examined by different techniques, including field emission scanning electron microscopy (FESEM), energy-dispersive X-ray spectroscopy (EDX), and X-ray diffractometer (XRD).

### 2.3. PHREEQC Modeling

A simplified thermodynamic simulation was employed using the geochemical computational modeling software PHREEQC v3 [45]. Adopting the parameters detailed in Table 1 for the produced water composition, the solution was specified as input representing the feed solution in a DCMD operation. Though the membrane’s physical properties could not be included due to the program’s limitation, water vapor extraction was simulated as a reaction to obtain water vapor pressure lowering in terms of ionic strength. The result was analyzed within the saturation index of potential scales output given by the same model and further compared to experimental results to evaluate the vapor pressure lowering effects and their concomitant permeate flux behavior.

### 2.4. Techno-Economic Analysis

The capital and operating costs of the DCMD-Cr process were evaluated on the assumption of a 100% of salt recovery from produced water, with several assumptions listed in Table 4 [46,47]. The use of two different heat resources was considered to heat PW from 20 °C to 60 °C: one is steam, and the other is waste heat (such as the heat from a water treater or natural gas that would otherwise be flared). The cost model was based on data from the reference and preliminary tests to estimate both capital and operating costs at a large scale [48].

The direct cost in this evaluation includes the crystallizer cost, calculated using the equation from Wang et al. for a continuous crystallizer [46]. PR is the crystal production rate of a continuous crystallizer in tons/day.

(3) $$PR=M_{cp}\times \frac{24}{1000}$$

where $M_{cp}$ is the mass of crystals produced in kg/h. In addition to this cost, the pre-treatment cost mentioned earlier in the assumptions is included to calculate the total capital cost for this section.

## 3. Results and Discussion

### 3.1. DCMD-Cr Performance Evaluation

#### 3.1.1. Permeate Water Flux and Salt Rejection

**Figure 2:**
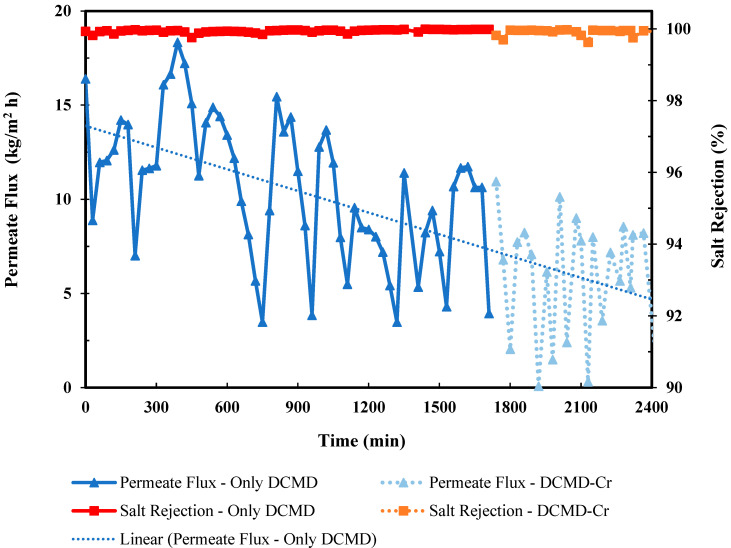
Desalination performance of the integrated DCMD-Cr process for ZLD of PW with an initial TDS of 156,700 mg/L.

The water flux over 40 h of continuous cyclic DCMD-Cr experiments is shown in Figure 2. Overall, the water flux decreases with time. During the standalone DCMD, PW was concentrated to its saturation point (280,000 mg/L). After each DCMD cycle, the feed TDS increased, leading to a corresponding decrease in water flux. This decline can be distributed to vapor pressure lowering cause by the increased salt concentration in the feed solution.

**Figure 3:**
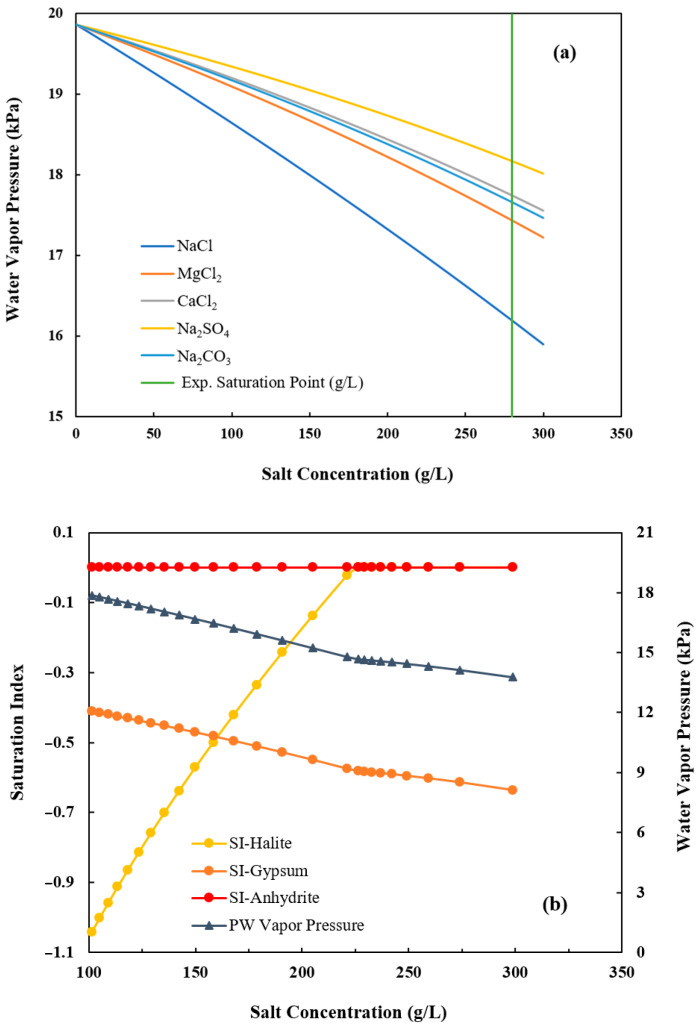
(**a**) Water vapor pressure of feed solutions at 60 °C with varying salt concentrations. The green line indicates the experimental saturation point of the feed solution at 280,000 mg/L. (**b**) Vapor pressure and saturation index of minerals in PW at different salt concentrations at 60 °C.

The water vapor pressure behavior was simulated using PhreeqC, a geochemical thermodynamic modeling program, and the results are also shown in Figure 3. The vapor pressure of all the salts decreases as the concentration rises. For example, as the NaCl concentration in the feed solution increases, the NaCl solubility decreases, leading to a rapid drop in water vapor pressure as water leaves the system as permeate flux. Once NaCl reaches saturation, it begins to precipitate as halite. Salts present in lower concentrations but with higher solubility remain dissolved until their solubility limits are also reached. In other words, once NaCl saturation is achieved (saturation index = 0), other dominant ions, such as Mg^2+^, Ca^2+^, SO_4_^2−^, and Cl^−^, begin to influence vapor pressure lowering, though at a slower rate than NaCl. This trend is consistent with the results in Figure 2, where the permeate flux declined to almost zero (0.072–0.032 kg/m^2^/h) at the end of DCMD, which can be explained by the vapor pressure lowering in Figure 3 where the vapor pressure difference along the membrane decreased to below 17 kPa (the value for pure water) at the saturation point.

The simulation may slightly underestimate the concentration at which salt precipitation and flux decline occur because it does not account for membrane properties, which also reduces the accuracy of the predicted vapor transfer rate. While the vapor pressure lowering behavior varies for different ions, the influence is reduced as the water content decreases during membrane distillation. In addition, the “caking” phenomenon, where crystallized salts form solid deposits on the membrane surface and cause membrane scaling, contributes to the rapid decline in permeate flux once the saturation point is reached.

Beyond the saturation point, the salts tended to form crystals, resulting in scaling on the membrane’s outer surface. With integrated crystallization, the flux was recovered for 30–60 min per cycle, which allows the operation to be extended until reaching the ZLD. On the other hand, the conductivity on the permeate side was kept below 10 µS/cm in most cycles, corresponding to a salt rejection above 99.8% during DCMD, and slightly decreased to 99.6% and 98.5% from the beginning of the coupling of crystallization due to high initial salt concentrations in the solution, until the salts were removed by the cooling transport mechanism and were close to reaching ZLD, respectively. The water flux oscillated between 10 kg/m^2^/h and 2 kg/m^2^/h during the DCMD-Cr cycles.

**Figure 4:**
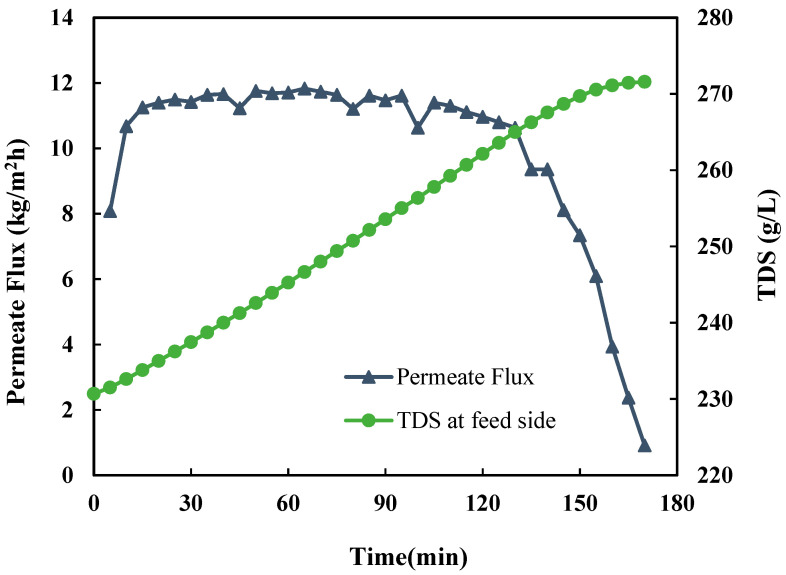
Permeate flux and salt rejection of DCMD during the cyclic DCMD-Cr process, with an initial TDS of 231,000 mg/L in the cycle.

Figure 4 presents the desalination performance of DCMD during a representative DCMD-Cr cyclic experiment with an initial TDS of 231,000 mg/L. The permeate flux remained stable at approximately 11.83 kg/m^2^/h as the TDS increased to 258,000 mg/L. However, it dropped sharply to just 0.91 kg/m^2^/h when the TDS approached 272,000 mg/L, which is near the saturation point of PW of 280,000 mg/L at 60 °C. For electrolyte solutions with strong water absorption, such as PW, which primarily contains NaCl along with other desiccant salts like CaCl_2_, the desalination performance is influenced by both membrane scaling and vapor pressure lowering. When the feed concentration reaches the saturation point, the water vapor pressure falls below 17 kPa, as shown in Figure 3, which is lower than the vapor pressure of pure water at 60 °C. In other words, the observed flux decline corresponds to reduced water activity, leading to lower vapor pressure on the feed side [29]. It was interesting to note that the increased TDS has a negligible effect before the concentration is close to reaching the saturation point. The permeate flux remains nearly constant until the feed solution approaches saturation, after which a sharp decline occurs. This can be attributed to the combined effects of vapor pressure lowering and the onset of surface crystallization (caking) due to supersaturation. It was also observed that the electrical conductivity of permeate remains less than 10 μS/m, indicating that there was no significant membrane wetting associated with the flux decline.

#### 3.1.2. Overall Water and Salt Recovery

**Figure 5:**
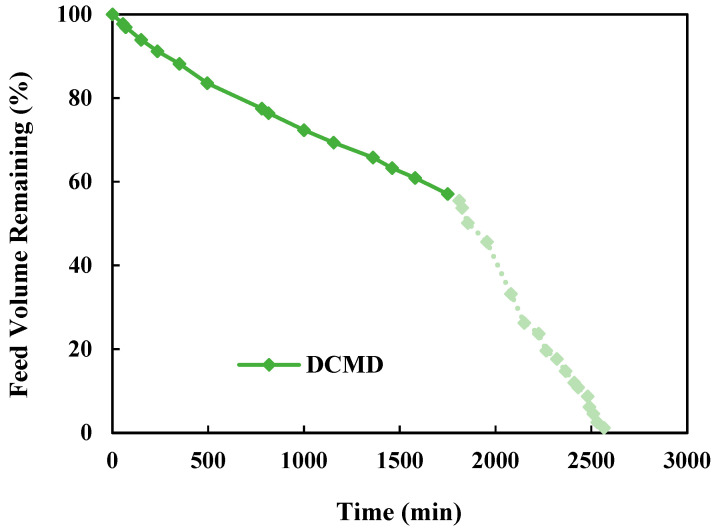
Reduction in feed solution volume during 40 h of continuous DCMD-Cr process for ZLD of real-produced water with an initial TDS of 156,700 mg/L.

**Figure 6:**
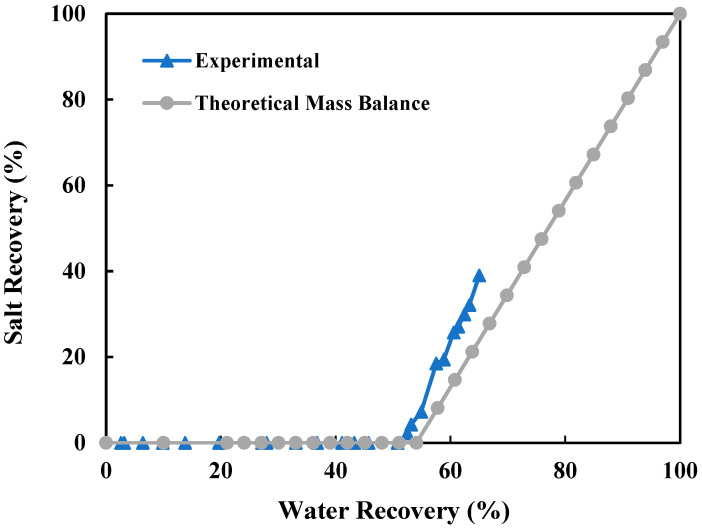
Relationship between salt recovery and overall water recovery during the 40 h of continuous DCMD-Cr process for ZLD of real-produced water with an initial TDS of 156,700 mg/L.

The pre-treated produced water with an initial TDS of 156,700 mg/L was directly used in a long-term continuous DCMD-Cr operation. For 68% of the operating time, a standalone DCMD was performed to recover water until the feed solution was close to its saturation point. During the remaining 32% of the time, the experiment shifted to simultaneous water and salt recovery to achieve ZLD. As shown in Figure 5, the standalone DCMD recovered a maximum of 42.94% of the total recoverable water. When the crystallization unit was integrated, the water recovery increased to 98.9%. Water recovery improved alongside salt recovery, as shown in Figure 6, where crystal formation began once water recovery reached approximately 45.8%, which matched well with the results from the theoretical mass balance.

The previously explained water flux decline leads to a slightly lower water recovery rate at the end of each cycle due to reduced trans-membrane vapor transport. After each cyclic DCMD-Cr operation, the membrane modules were regenerated with DI water, which helped maintain a consistent water recovery performance even after crystal formation. As shown in Figure 5, the initial water recovery was about 5% per cycle, and it dropped with the operating time, attributed to the accumulated salt deposits on the membrane surface, which thus reduced the water flux. However, the crystallization process mitigated this reduction, allowing for a slower decline in desalination performance by significantly inhibiting the membrane scaling when concentration approached the saturation point.

#### 3.1.3. Salt Characterization

**Figure 7:**
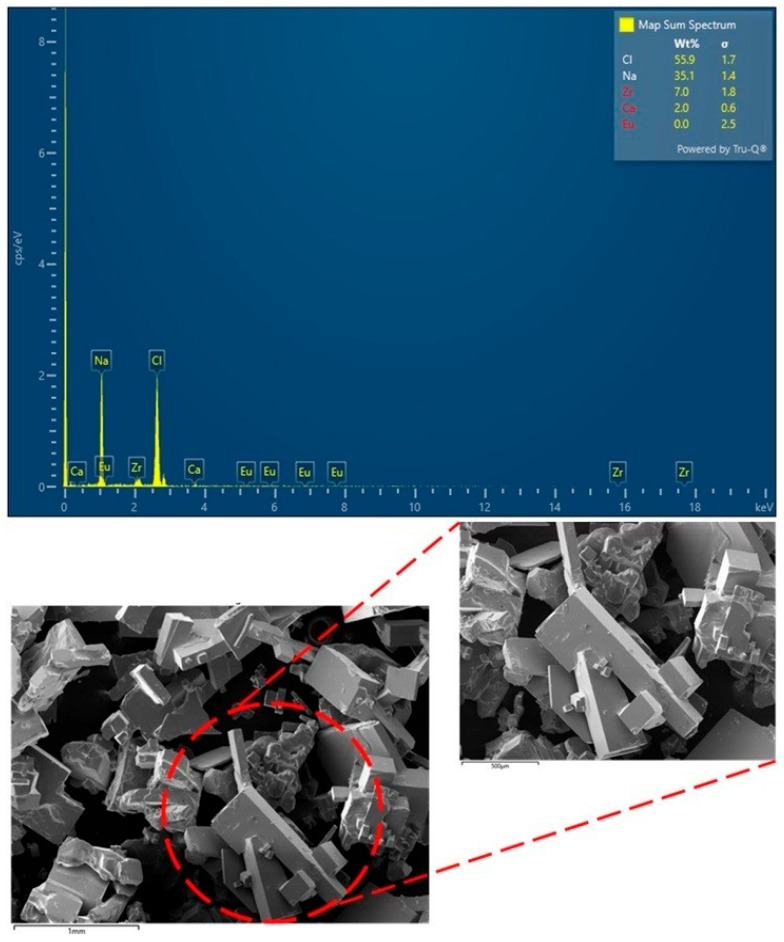
SEM images and EDS spectra of recovered crystals at the end of the DCMD-Cr cyclic experiments, with 88% water reduction.

The crystals in the crystallization unit at the end of every DCMD-Cr cyclic experiment were collected and characterized. Since all the crystals show highly similar morphology and composition, as a representative, the crystals collected at the end of the cycle with an 88% feed volume reduction are shown in Figure 7. It indicates that majority crystals are sodium chloride, as illustrated by the cubic shape with small deviations [37]. Moreover, the large, prism-like crystals can be attributed to calcium sulfate. The recovered solids are predominantly rectangular-cuboid halite with a characteristic length of ≈1 mm and a width/thickness of ≈0.5 mm. This sub-millimeter morphology is significantly smaller than the millimeter-to-centimeter halite typically obtained in slow evaporative crystallizers, indicating effective crystal formation under our cooling-based operation. Smaller crystal size is generally beneficial for the integrated DCMD-Cr process for several reasons [12]: (i) a higher surface-area-to-mass ratio promotes more uniform growth and dissipates local supersaturation, suppressing crust formation and caking on the MD module; (ii) a larger number of particles at a given solids production rate favors distributed nucleation and shorter induction time, which stabilizes the flux when the feed approaches saturation.

**Figure 8:**
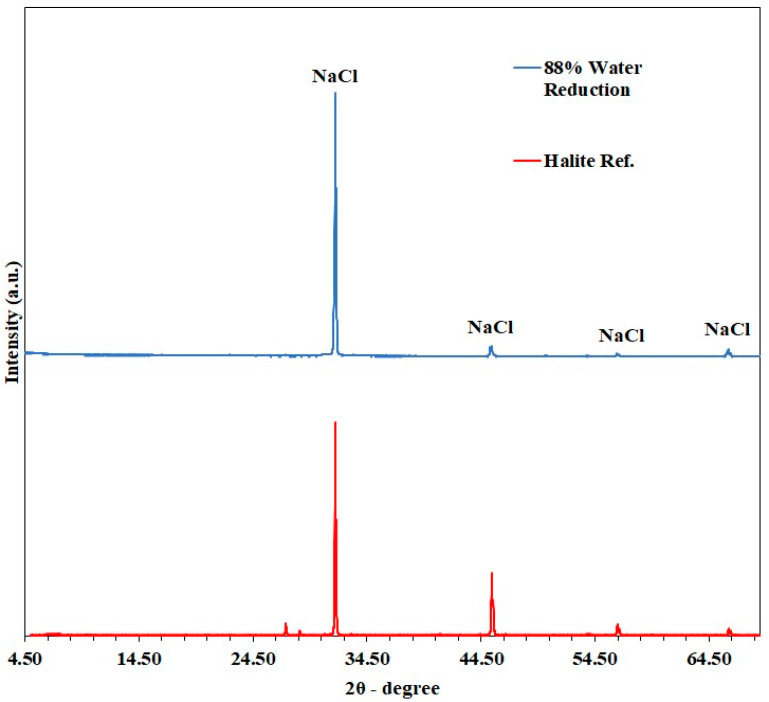
XRD spectra of recovered crystals at the end of the DCMD-Cr cyclic experiments, with 88% water reduction.

The XRD results in Figure 8 confirm the compositions of crystals, as indicated by the consistent spectra of the crystal sample with Halite as the reference. The lack of calcium sulfate and other salts might be attributed to the detection limit of 5% for XRD analysis.

### 3.2. Optimization and Scale-Up

**Figure 9:**
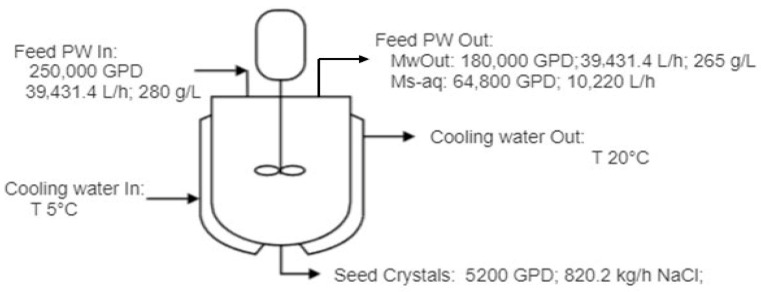
Mass balance analysis of sodium chloride in the cooling-based crystallization unit during integrated DCMD-Cr cyclic experiments.

The techno-economic analysis was conducted based on the feed water reduction in Figure 6, which shows that nearly 100% of the water had been recovered from PW. The cost model is based on a 500,000 GPD (78,846 kg/h) plant capacity, including PW pre-treatment, DCMD, and crystallization. Figure 9 shows the mass balance in the crystallization unit, indicating that around 5200 GPD (820 kg/h) of crystals were recovered from the crystallization once the mother liquor (concentrated PW from DCMD) was saturated at a T_cr_ of 20 °C, with a solubility of 360,000 mg/L for NaCl. The concentration of the concentrated PW was reduced to 265,000 mg/L while keeping the cooling temperature at around 20–25 °C. With an initial TDS of 156,700 mg/L in the produced water, the NaCl crystal recovery was 16 g/kg. In comparison, Luo et al. combined membrane distillation with SHFCC and reported a recovery of 64 g of NaCl crystals per kg of feed containing 23 wt.% NaCl [12].

The techno-economic analysis for the DCMD-Cr process is summarized in Table 5. The results indicate that the operating cost is the major cost for DCMD-Cr operation, mainly attributed to the energy consumption for cooling in the crystallization unit. The PW treatment cost is USD 1.41 per barrel of clean water produced using steam, and the cost can be reduced to USD 0.64 per barrel of clean water produced if the heat in the heat treaters or the flared natural gas can be integrated as the heat resource. It is worth noting that the revenue varies depending on the type of recovered salts. Considering the industrial-grade sodium chloride price of USD 50 per ton, and a crystals production rate at around 19,684 kg/d, as shown in Figure 9, the revenue from sodium chloride is USD 0.14 per barrel, which reduces the total cost of DCMD-Cr to USD 1.27/bbl and USD 0.50/bbl for the scenarios of using steam and waste heat, respectively.

## 4. Conclusions

Direct contact membrane distillation and crystallization processes were integrated for zero liquid discharge (ZLD) of a high-salinity produced water by simultaneous recovery of clean water and salt. The two processes show promising synergies that the integrated crystallization significantly increased the water recovery from 42.0% to 98.9% in DCMD, while DCMD promotes crystal formation and salt precipitation in crystallization. The integrated process shows a stable salt rejection of 99.8% and 98.5% in DCMD and integrated DCMD-Cr processes, respectively. The water flux is largely determined by the overall water recovery and the associated vapor pressure lowering phenomenon. Membrane scaling was observed due to the deposition of sodium chloride crystals on the membrane surface, but it can be easily remediated in the cyclic DCMD-Cr process. The preliminary techno-economic analysis results indicate that the cost for ZLD of PW is dominated by the cooling-based crystallization operating cost. The use of renewable heat resources and salt recovery from the crystallization unit can significantly improve the economic feasibility of the DCMD-Cr process by reducing the cost to USD 0.50 per barrel.

Future work should focus on the long-term stability of the membranes in terms of changes in wettability, mechanical stability, and regeneration ability. The effects of operating parameters on crystallization performance should be studied to maximize the recovery efficiency and crystals purity. The minerals in the crystals other than sodium chloride should be thoroughly identified. Specifically, potential valuable resources, such as critical minerals and rare earth elements, can be selectively recovered through the integration of other separation processes.

## Figures and Tables

**Table 1:** Summary of real-produced water desalination using MD-Cr.

MDC	Crystallization		Membrane Characteristics		Feed				Permeate				Results				References
	Method	Temp. (°C)	Material	Pore Size (µm)	Type	Temp. (°C)	TDS (ppm)	Flow Rate	Type	Temp. (°C)	TDS (ppm)	Flow Rate	Permeate Flux	ZLD	Type of Salt Recovered	Total Cost	
DCMD-Cr	Cooling	20	PVDF- HF	0.27	Oilfield PW	60	156,700	100 rpm	DI	20	-	40 rpm	1.5–7.5 kg/m^2^h	✓	NaCl	1.41 0.64 (w/waste heat) USD/barrel	**This study**
DCMD-Cr	Evaporation	-	PP and PVDF -HF	0.2, 0.23	Oilfield PW	35 45 55	248,000	150–250 mL/min	DI	10	-	70 mL/min	1–3.4 L/m^2^h	✕	NaCl	(w/salt sale) 1.52, 1.24 USD/m^3^	[37]
DCMD-Cr	Evaporation and Cooling	40	PTFE-FS	0.22	Synthetic SGPW	60	150,000	CFV 0.25 m/s	DI	20	-	CFV 0.25 m/s	-	✓	BaCl_2_, CaCO_3_, NaCl		[38]
DCMD-Cr	Cooling	10	PP-HF	0.17	Oilfield PW	55–75	116,800	-	DI	-	-	-	1.5–2.5 kg/m^2^h	✕	NaCl	-	[39]
DCMD-Cr	Cooling	15	PP-HF	0.2	PW	38	240,000	200 L/h	DI	25	-	-	2.5 L/m^2^h	✕	-	-	[11]
DCMD-Cr	Evaporation and Cooling	30–50	PP-HF	0.22	SGPW	60	30,000	1.2–3.6 L/min	DI	20	-	CFV 0.1–0.3 m/s	2.5 kg/m^2^h	✓	NaCl CaCO_3_	-	[2]
OMD-Cr	Evaporative	-	PTFE-PES	-	CaCO_3_ NaCl Solution	77–80 64–78	70–72 70–76	4–8 L/min	DI	70–72 70–76	-	4–8 L/min	6–16 1–30 kg/m^2^h	✓	CaCO_3_ NaCl	1.09/m^3^	[17]
VCMD-Cr	Cooling	20	PP-HF	0.18	Radioactive wastewater	20–70	500–110,000	41.8 L/h	Vacuum	-	-	0.97 atm	6.2–5.5 kg/m^2^h	✕	Boric Acid	-	[25]
DCMD-Cr	Cooling	-	PP-HF	0.2	Anodizing wastewater	38–56	887,000	21.6 L/h	DI	15	-	0.8–1.7 m/s	1.29–3.86 kg/m^2^h	✓	Na_2_SO_4_	-	[43]
DCMD-Cr	Evaporative and Seeding	-	PP	0.2	Sludge dewatering reject	45–65	1391– 2345	20 L/h	-	-	-	-	0.0013–0.0105 kg/m^2^h	✕	Phosphorous	-	[26]
DCMD-Cr	Evaporation	60	PVDF	0.22	Seawater	60	65,000	200–1250 mL/min	DI	20	-	-	3.8 kg/m^2^h	✕	-	-	[18]

**Table 2:** Ionic composition in produced water.

Property	Species	Original PW Values
Cations (ppm)	Sodium Na^+^	49,958
	Potassium K^+^	893
	Magnesium Mg^2+^	1132
	Calcium Ca^2+^	10,724
	Ammonium NH_4_^+^	1128
	Lithium Li^+^	28
Anions (ppm)	Chloride Cl^−^	96,560
	Bromide Br^−^	1094
	Sulfate, SO_4_^2−^	544
Alkalinity (ppm)	HCO_3_^−^	98
Total Dissolved Solids (TDS) (ppm)		156,700
pH		7.24

**Table 3:** Membrane and module properties.

Properties	Value	Units
Porosity	80.6 ± 0.4	%
Thickness	135 ± 2	µm
Diameter Outer	1088 ± 2	µm
Diameter Inner	767 ± 2	µm
Pore Size	0.27 ± 0.02	µm
Bubble Point Pore Size	0.40 ± 0.02	µm
Configuration	Hollow Fiber (HF)	-
Module Length	22	cm
Number of Fibers in the Module	6	-

**Table 4:** General scale-up assumptions.

	Value	Units
Plant Capacity	500,000 (78,846)	GPD (kg/h)
Plant Lifetime	30	Year
Plant Availability	90	%
Produced Water, Initial TDS	100,000	mg/L
Produced Water, Concentrate TDS	280,000	mg/L
Recovered Product Water	<50	mg/L
Water Recovery	90	%
Membrane Cost	20	USD/m^2^
Membrane Replacement	20	%/year
Pre-Treatment	80	USD/m^3^/day
Electricity Cost	0.069	USD/kWh
Steam Price	0.008	USD/kg

**Table 5:** Scale-up normalized capital and operating cost for the two scenarios.

Scenario	Capital Cost (USD/bbl.)		Operating Cost (USD/bbl.)		Total Cost (USD/bbl.)
	MD	Crystallization	MD	Crystallization	
(1)	0.08	0.04	0.79	0.50	1.41
(2)	0.08	0.04	0.02	0.50	0.64

## Data Availability

The original contributions presented in this study are included in the article. Further inquiries can be directed to the corresponding author.
